# Evolution of the muscular system in tetrapod limbs

**DOI:** 10.1186/s40851-018-0110-2

**Published:** 2018-09-20

**Authors:** Tatsuya Hirasawa, Shigeru Kuratani

**Affiliations:** 1 Laboratory for Evolutionary Morphology, RIKEN Center for Biosystems Dynamics Research (BDR), 2-2-3 Minatojima-minami, Chuo-ku, Kobe, Hyogo 650-0047 Japan; 2 Evolutionary Morphology Laboratory, RIKEN Cluster for Pioneering Research (CPR), 2-2-3 Minatojima-minami, Chuo-ku, Kobe, Hyogo 650-0047 Japan

**Keywords:** Development, Evolution, Homology, Fossils, Regeneration, Tetrapods

## Abstract

While skeletal evolution has been extensively studied, the evolution of limb muscles and brachial plexus has received less attention. In this review, we focus on the tempo and mode of evolution of forelimb muscles in the vertebrate history, and on the developmental mechanisms that have affected the evolution of their morphology. Tetrapod limb muscles develop from diffuse migrating cells derived from dermomyotomes, and the limb-innervating nerves lose their segmental patterns to form the brachial plexus distally. Despite such seemingly disorganized developmental processes, limb muscle homology has been highly conserved in tetrapod evolution, with the apparent exception of the mammalian diaphragm. The limb mesenchyme of lateral plate mesoderm likely plays a pivotal role in the subdivision of the myogenic cell population into individual muscles through the formation of interstitial muscle connective tissues. Interactions with tendons and motoneuron axons are involved in the early and late phases of limb muscle morphogenesis, respectively. The mechanism underlying the recurrent generation of limb muscle homology likely resides in these developmental processes, which should be studied from an evolutionary perspective in the future.

## Background

The fossil record reveals that the evolutionary rate of vertebrate morphology has been variable, and morphological deviations and alterations have taken place unevenly through history [1–5]. Sporadic geneses of new homologies, or units of evolutionary alterations, reflect this uneven evolutionary tempo. A synthesis of paleontology and evolutionary developmental biology may help to increase our understanding of how morphological homologies sporadically arise and why they are conserved in subsequent generations. However, in most cases, only post-embryonic morphology is observable in fossils, making it difficult to attribute observed evolutionary changes to certain developmental changes.

In the vertebrate body, skeletal muscles are connected to specific sites of connective tissues, such as bones, and these connections are generally unchanged after their initial formation. Thus, evolutionary changes in muscle connections, which can also be observed in fossil bones, correspond to changes in morphogenetic process, unlike other morphological characters that may change during growth. Skeletal muscles thus exhibit clear advantages for the integration of paleontology and evolutionary developmental biology. This paper aims to summarize the current understanding of the evolution and development of skeletal muscles in the hopes of providing a basis for future studies. In particular, from the perspective of the role of developmental constraints in evolution [6], we focus on forelimb muscles, which were functionally diversified in tetrapod history. In regards to the interplay between developmental and functional constraints that shapes evolution, the conventional approach to modes of evolution [1] has addressed functional aspects, or adaptations, but has too often neglected developmental constraints as black boxes. We seek to remedy this deficit by suggesting a new framework for incorporating developmental constraints into researches on modes of evolution.

## Evolutionary history of tetrapod limb musculoskeletal systems

In comparative anatomy, the homology of forelimb muscles among extant tetrapod species is identifiable based on gross anatomy, such as the connections between these muscles and bones or innervations, and the same set of names has been applied to different tetrapod classes [7–16], although there have been a few misidentifications in classical papers, e.g., for turtles [17]. Since the topographical relationships among limb muscles and their attachment sites are rather well conserved in extant tetrapods, reconstructions of muscles on the limb skeletons of fossil tetrapods has been achieved [18–24]. However, determining the one-to-one homology between tetrapod limb muscles and fish fin muscles has been more difficult [20, 24–27]. Extant tetrapods possess as many as 30–40 individual muscles with specific names in their forelimbs, while extant fishes possess fewer than 10 descriptive pectoral fin muscles [26, 28, 29]. Clearly, substantial new homologies in the musculature were acquired during the fin-to-limb transition.

**Fig. 1:**
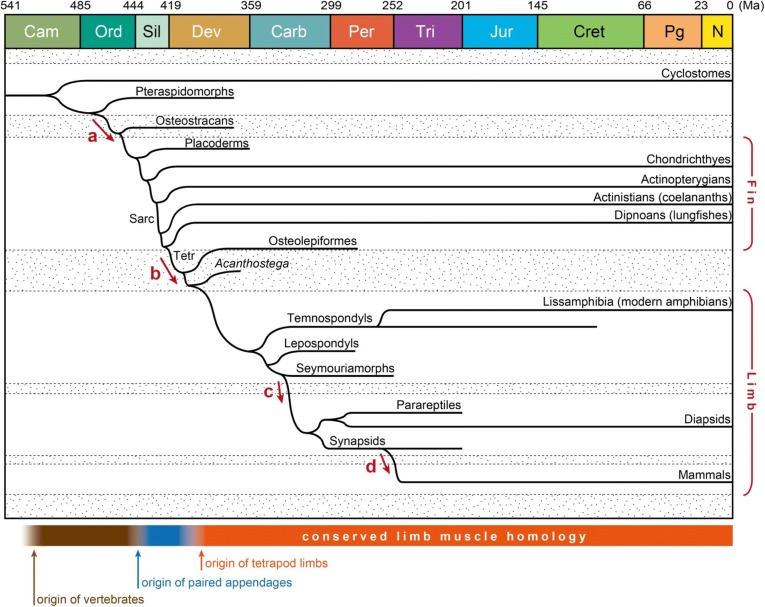
Evolution of the limb muscles on the time-calibrated phylogenetic tree. **a** Acquisition of paired fins. **b** Establishment of the limb muscles and brachial plexus. **c** Loss of aquatic larval stage and regeneration capability. **d** Evolution of the diaphragm from a shoulder muscle. The stippled areas stand for putative transitional forms separating “grades” in fin/limb muscle evolution. The bar in the bottom shows the timescale of the limb muscle evolution. Sarc Sarcopterygia, Tetr, Tetrapodomorpha

The evolution from fin muscles to limb muscles occurred deep in time (Fig. 1; the numerical values for ages follows the Geological Time Scale v.4.0 [30]). In the geological time scale, vertebrates first emerged in the fossil record around 520 million years ago [31–33], and the earliest fossil occurrences of paired fin-bearing gnathostomes are in the Early Silurian, 444–433 million years ago [34, 35]. The osteostracans, a stem-group of the gnathostomes, possessed only pectoral fins, but the endoskeletal elements were already present in their pectoral fins [36], suggesting that the fin musculoskeletal system originated in the common ancestor of osteostracans and crownward lineages (Fig. 1, arrow a).

Pelvic fins evolved in placoderms and crown-group gnathostomes [37], and from the latter, sarcopterygians evolved 423 million years ago (Ludlow Epoch of the Silurian) [38] (Fig. 1). Tetrapodomorphs evolved as a clade within the Sarcopterygia (Fig. 1), specifically sharing the last common ancestor with dipnoans (lungfishes) [39, 40]. Analysis of fossil trackways [41, 42] has suggested that limb-bearing tetrapods first walked on the ground around 400 million years ago, and body fossils of limb-bearing tetrapods have been discovered from the stratum of 375 million years ago [43, 44], providing physical evidence for the minimum age of the limb-bearing tetrapod history.

During the evolutionary transition from pectoral fin to forelimb, the ulna became as large as the radius, and the articular facets of the elbow and wrist joints turned, enabling the support of the body on a substrate [45–48], although the mobility of these joints was limited in the early limb-bearing tetrapods [49].

As for muscles, it is likely that the major morphological and topographical transitions took place concomitantly with the skeletal evolution, giving rise to the elbow and wrist joints of the forelimb. Indeed, the cross-section shape of the humerus and some muscle attachment sites on its surface in a basal limb-bearing tetrapod [24, 50] are consistent with this assumption. Thus, ancestral limb muscles had already emerged within the first 30% of the total history of vertebrate evolution (~ 520 million years). In addition, whereas the fin-to-limb transition took place in a short period of the evolution of paired appendage (Fig. 1, arrow b), limb muscles were not significantly modified for around 85–90% of the whole paired appendage history (Fig. 1). Considering the period of time to be a proxy for the number of generations, the long absence of evolutionary deviation for limb muscles represents strong empirical evidence of both the robustness of limb muscle development and the singularity of its evolutionary origin.

Despite the conservation of limb muscle homology, the development of limb muscles is variable in timing and in the environment surrounding the progenitor cells. In amniotes, limb muscles develop almost in parallel with other skeletal muscles during embryonic development, and become functional before birth, whereas in many species of extant amphibians, the limbs and their muscles develop during larval stages [51–53]. Such relatively delayed development of limb muscles in amphibians has repeatedly led to the conclusion that these limb muscles are of lateral plate mesodermal origin [54, 55] unlike those of amniotes, which are of somitic origin [56–58]. However, in the current understanding, the limb muscles of amphibians are also of somitic origin [59–61]. In addition, concomitant with a unique Hox gene expression pattern [62, 63], the developmental sequence of limb skeleton [64, 65] and muscles [66] in urodele amphibians is opposite to that in amniotes and anuran amphibians. Moreover, extant amphibians, especially urodeles, show high capabilities of regeneration of limb musculoskeletal systems [67–69]. In these amphibians, limb muscle homology is recurrently formed both in normal development and in regeneration, providing further evidence of the robustness of limb muscle development.

Extant amphibians consist of only a fraction of several anamniote tetrapod lineages, and the phylogenetic position(s) of extant amphibians remains a matter of controversy. In one hypothesis, extant amphibians are all included in a single clade, the Lissamphibia, which evolved from the Temnospondyli, whereas the Amniota evolved from another clade, from which the extinct Seymouriamorpha and Lepospondyli also branched off [39, 70–72] (Fig. 1). An alternative hypothesis assumes the lepospondyl affinity of extant amphibians [73]. In both hypotheses, the data on these fossil anamniote taxa provide insights into the ancestral condition of the limb development.

Many stem anamniotes (basal temnospondyls, seymouriamorphs and lepospondyls), similarly to lissamphibians, had an aquatic, gill-bearing larval or juvenile stage [74–76]. Thus, the common ancestor of crown-group tetrapods likely had an aquatic larval/juvenile stage also. Although metamorphosis, which involves rapid morphological reorganization, evolved within the lissamphibian stem lineage [4, 77], it is possible that limb muscles developed in post-embryonic remodeling, at some point during the free-swimming larval or juvenile period in fossil anamniotes including the ancestors of amniotes, as suggested by data of basal temnospondyls [78–80], lepospondyls [80, 81], and the fin-bearing tetrapodomorph *Eusthenopteron* [82]. It is worth considering the possibility that the post-embryonic development of limb muscles seen in extant amphibians represents the ancestral state for tetrapods. Additionally, the possibility that the major evolutionary changes in developmental sequence could only have occurred in the early evolution of tetrapods [4] deserves consideration from the perspective of temporal change of evolvability.

The fossil record provides some indication for the development of the forelimb in the stem temnospondyls proceeding from the radial to the ulnar sides, as in the urodeles [83, 84]. Accordingly, the difference in developmental sequence between urodele and anuran/amniote limbs likely reflects two or more evolutionary changes in this developmental signature, rather than urodele synapomorphy.

Extant urodele amphibians are able to regenerate limb muscles [85, 86]. Similar regeneration capabilities have been recognized in fossils of the stem temnospondyls and lepospondyls [84]. In addition, a recent study demonstrated that lungfishes, the sister group of the tetrapodomorphs, regenerate fins in a process similar to that in urodeles, by deploying gene regulatory networks that shared, at least in part, with those of urodeles, which suggests that the capacity for regeneration is plesiomorphic to tetrapodomorphs [87] (Fig. 1, arrow c). Although this regenerative competence was secondarily lost in amniotes and anurans, a common mechanism for recurrently generating limb muscles may underlie both development and regeneration. Future research on limb muscle regeneration may lead to a better understanding of the developmental mechanisms underlying limb muscle homology and its evolutionary origins.

## Brachial plexus as an evolutionary novelty

**Fig. 2:**
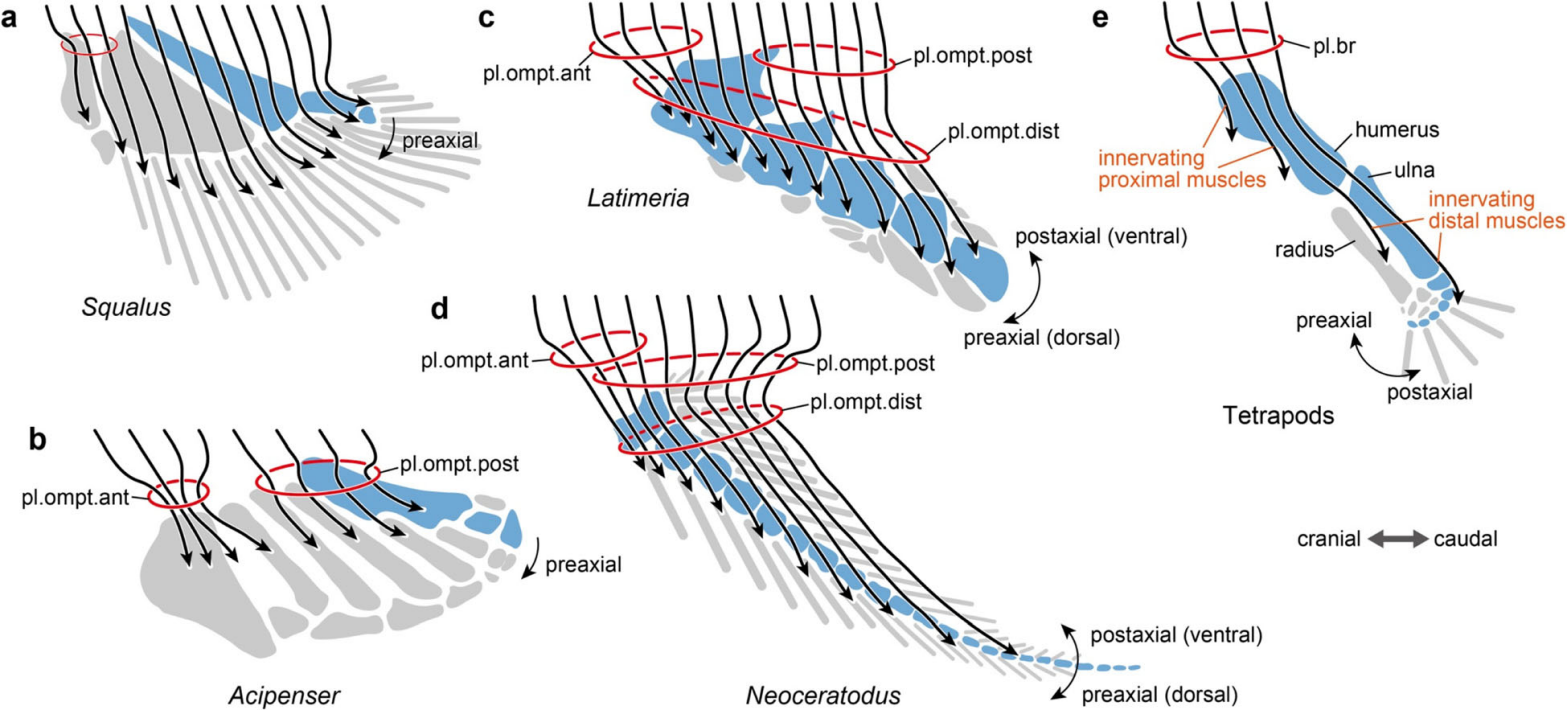
Comparison of innervation patterns of pectoral fin/forelimb muscles according to Fürbringer’s theory. **a** *Squalus* (shark, elasmobranch chondrichthyes). **b** *Acipenser* (sturgeon, non-teleost actinopterygian). **c** *Latimeria* (coelacanth, actinistian sarcopterygian). **d** *Neoceratodus* (dipnoan sarcopterygian). **e** Tetrapods. Red circles indicate positions of the plexus (in *Squalus,* the anastomosis). Arrows shows spinal nerves joining pectoral fin/forelimb muscle innervations, and their respective innervating portions (muscles) are simplified as paths of arrows, according to Fürbringer [88]. Skeletal elements of the metapterygial axis are colored in blue, and the other (preaxial or postaxial) skeletal elements in gray. pl.br, *plexus brachialis*, pl.ompt.ant *plexus omopterygius anterior*, pl.ompt.dist *plexus omopterygius distalis*, pl.ompt.post *plexus omopterygius posterior*. **a** b and **d** are based on Braus [95]. **c** is based on Millot and Anthony [102]. Metaptarygial axes are based on Shubin and Alberch [64]

Fürbringer once emphasized that a motor nerve and its innervating skeletal muscle constitute a unitary structure (*neuromotorische Apparate*) [88]. In this scheme, the homology of limb muscles is linked with that of motor nerves, which extend from the central nervous system to the skeletal muscle, often forming anastomoses before innervation (Fig. 2).

Tetrapod forelimb muscles are innervated by nerves that branch off from the brachial plexus [25, 89–93]. In elasmobranchs, pectoral fin muscles are innervated by the main trunks of the spinal nerves, which lack extensive anastomoses [89, 94–97] (Fig. 2a). In the actinopterygians and non-tetrapod sarcopterygians (i.e., coelacanths and lungfishes), the fin muscles are innervated by plexus-forming nerves (Fig. 2b–d). The plexuses of these osteichthyan fishes are composed of both occipital and spinal nerves [94, 95, 98].

According to the previous anatomical descriptions, a spectrum of complexity of anastomoses between fin muscle-innervating nerves is recognizable in osteichthyan fishes. However, most fish taxa show the shared feature that the plexus of nerves innervating the pectoral fin muscles can be subdivided anteroposteriorly into two parts; namely, the *Plexus omopterygialis anterior* and *Pl. omopterygialis posterior*, although relatively inconspicuous anastomoses exist between them [95]. In embryonic development of the Australian lungfish (*Neoceratodus forsteri*), these two plexuses develop separately across the first rib [99]. In general, *Pl. omopterygialis anterior* is more elaborated than *Pl. omopterygialis posterior*. In some actinopterygian species, *Pl. omopterygialis posterior* is nothing more than a series of connections between nerves running independently [95, 100].

Besides the commonality of the two subdivided plexuses, there is a difference in plexus formation between the actinopterygian and sarcopterygian fishes. In sarcopterygian fishes, pectoral fin muscles develop distally to span the distal skeletal joints through tendinous insertions, whereas in actinopterygians, muscles cover only the proximal portion of the pectoral fin [26, 27, 95, 101]. Concomitant with the differences in muscle distribution, unlike actinopterygians (Fig. 2b), sarcopterygian fishes possess an additional nerve plexus distal to *Pl. omopterygialis anterior* and *posterior* within the muscles of the pectoral fin (Fig. 2c, d). Braus named this distal plexus as *Pl. omopterygialis distalis* [95] in his description of the Australian lungfish (*N. forsteri*). A comparable plexus is also identifiable in the extant coelacanth (*Latimeria chalumnae*) [102].

For a wide range of tetrapod taxa, topographical patterns of brachial plexuses have been described in detail [12–16, 103–108]. Although inter- and intraspecific [109] variations exist, a comparable branching pattern is recognizable in tetrapod brachial plexuses; this has been used for homologizing forelimb muscles [25, 88]. Unlike pectoral fin muscles in fishes, forelimb muscles in tetrapods are innervated by only seven or fewer spinal nerves. In amniotes, brachial plexuses typically consist of four spinal nerves at the cervico-thoracic boundary of the axial musculoskeletal system [110]. Most limb muscles are innervated by nerve fibers composed of two or more roots of the plexus, or spinal nerves [88]. A set of these features is shared exclusively among tetrapods, suggesting that the brachial plexus evolved as a new unit of homology, or an evolutionary novelty.

Regarding the evolutionary origin of the brachial plexus and forelimb muscles, Fürbringer [88] once presented a hypothesis, which was supported by Braus [95] but has long since been forgotten. Fürbringer [88] proposed that in tetrapods most proximal limb muscles are innervated by nerves of anterior (preaxial) roots of the brachial plexus, whereas most distal limb muscles are innervated by nerves of posterior (postaxial) roots (Fig. 2e). In addition, he noted that the width of the appendage, in terms of number of associated spinal nerves (or somites), became narrowed at the fin-to-limb transition. Based on these observations, he formulated an evolutionary scenario from fin to limb: concomitant with the narrowing of the appendage, the antero-posterior axis of the innervation pattern and accompanying musculature in fish fins was shifted to the proximo-distal axis in tetrapod limbs, and this change brought about the dissolution of the segmentation pattern of spinal nerves and musculature. Although Fürbringer [88] did not specifically discuss skeletal homology, his theory is consistent with the evolutionary change in orientation of the metapterygial axis of skeletal elements across the fin-to-limb transition [64] (Fig. 2).

## Migratory muscle precursors

**Fig. 3:**
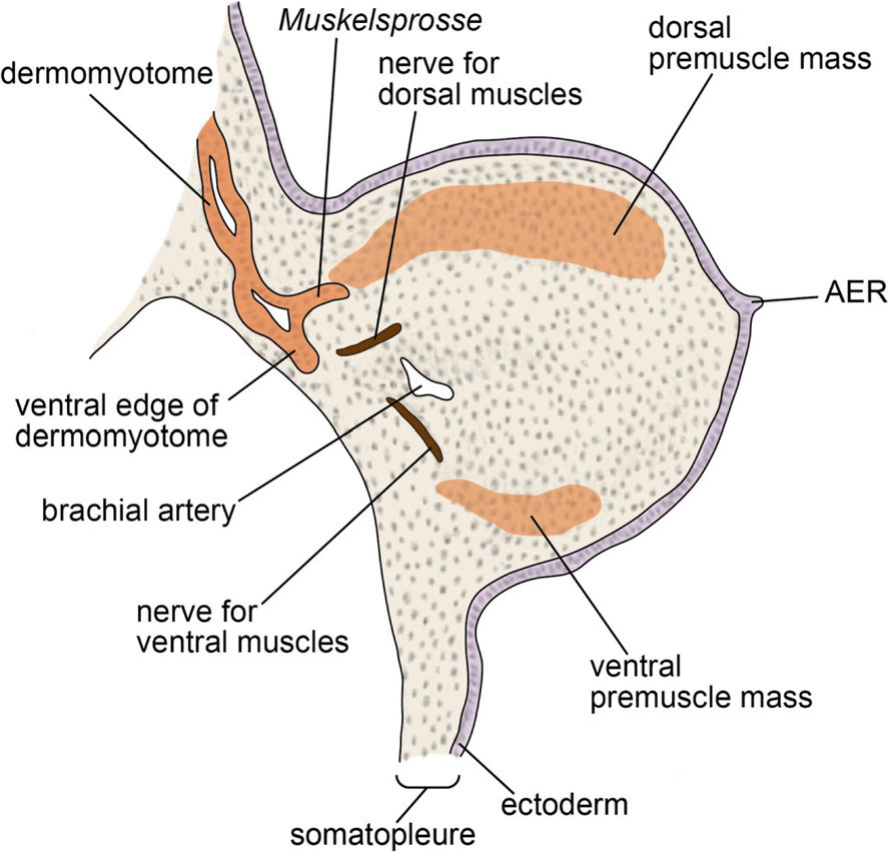
Dorsal and ventral premuscle masses in the forelimb bud of *Lacerta viridis* (European green lizard). Modified from Corning [112]. AER apical ectodermal ridge

Since the late nineteenth century, detailed observations of histological sections have been conducted for studying the development of limb muscle. Early scholars found that, in amniote embryos, limb muscles develop from migrating somitic cells, which are secondarily released from the segmentation pattern of somites [100, 111–114]. According to these observations of amniote embryos, the ventrolateral ends of the dermomyotomes, which extend toward the base of the limb bud, lose their epithelial structures at a certain developmental stage, and subsequently such de-epithelialized cells become dissolved into the mesenchyme of the limb bud (Fig. 3). This dissolution contrasts with the ventrally extending process of the dermomyotome, which forms the body wall muscles in amniotes. Within the limb bud, these migrating somitic cells can be distinguished histologically from the surrounding mesenchymal cells by their relatively large size of nucleus, possibly reflecting a less defined transcription pattern in chromatin dynamics [115], and they form cell masses, called “premuscle masses” [55, 113] or “muscle masses” [116, 117], before myogenesis. In the early phase of migration and proliferation of the de-epithelialized dermomyotome-derived cell population, there are two—dorsal and ventral—premuscle masses within the limb bud (Fig. 3), and these premuscle masses are not distributed in the body wall, where the shoulder girdle develops [114]. As development proceeds, the medial portions of the premuscle masses expand toward the body wall. In other words, the premuscle masses initially intrude laterally into the limb bud but not the body wall, and then a part of the premuscle masses intrudes medially into the body wall [113, 114, 118]. A century later, this phenomenon was confirmed and termed the “in-out” mechanism [119]. Through this mechanism, the muscles spanning the limb skeleton and trunk (i.e., the pectoralis and latissimus dorsi muscles) develop [113, 114, 118, 119]. In contrast, the muscles connecting the girdle skeleton with the trunk (i.e., the rhomboideus and serratus muscles) develop as part of the body wall muscles [114, 118–120]; thus, they have often not been classified as limb muscles [118]. In the above classification, true limb muscles develop from the premuscle masses that cancel the segmentation pattern and migrate to the limb bud, at least temporarily.

**Fig. 4:**
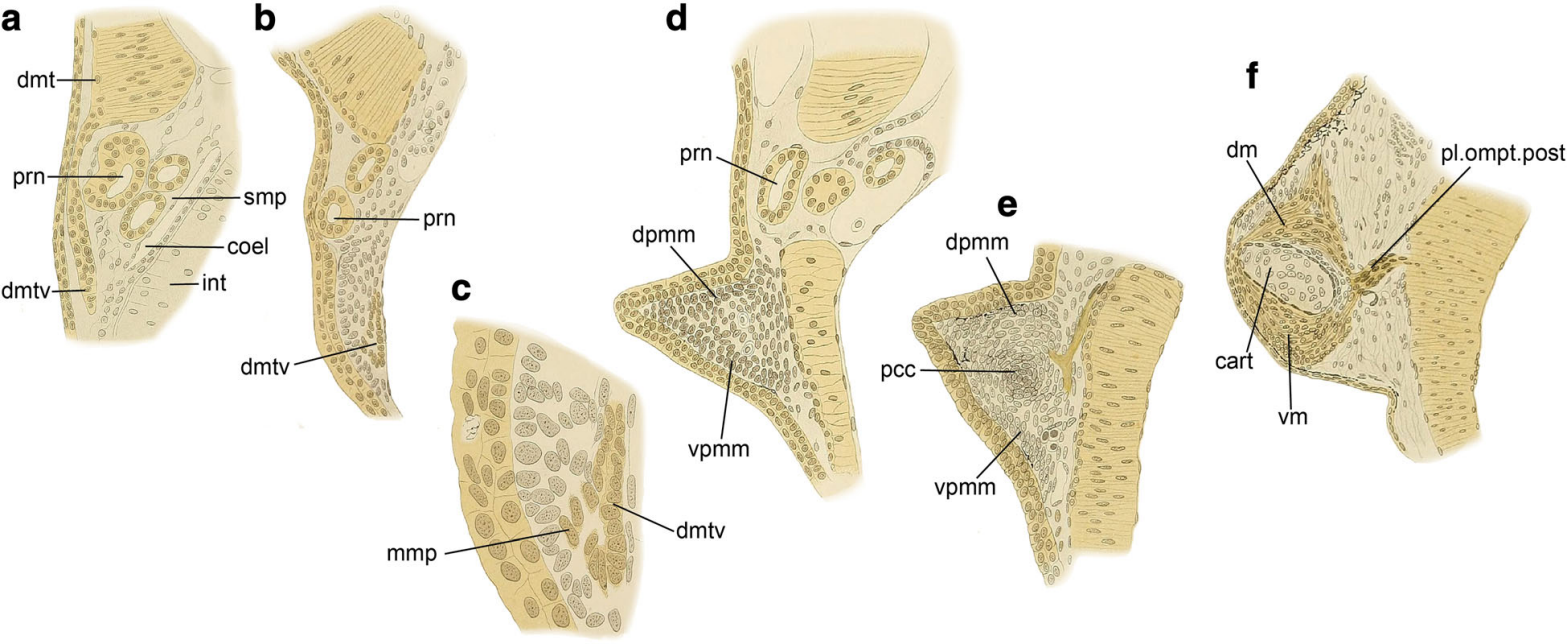
Development of the pectoral fin muscles in *Neoceratodus forsteri* (Australian lungfish) [99]. **a** Ventral process of the dermomyotome extending ventrally across the pronephric ducts at Stage 42. Note the dermomyotome in *N. forsteri* is not segregated from the myotome, unlike in amniotes. **b** Ventral process of the dermomyotome separated from the dorsal dermomyotome at Stage 43+. **c** Enlarged image of the ventral process of the dermomyotome in (**b**). At this stage, the migratory muscle precursors (MMPs) are delaminated from the lateral lamina of the ventral process of the dermomyotome, showing a similarity with amniote limb muscle precursor cells. **d** Dorsal and ventral premuscle masses at Stage 44+. At this stage, individually migrating cells are distributed in the dorsal and ventral parts of the fin bud in *N. forsteri*, like in amniotes (see Fig. 3). **e** Dorsal and ventral premuscle masses at Stage 46. **f** Onset of myofibers of the dorsal and ventral muscles of the pectoral fin at Stage 48. cart, cartilage; coel, coelom; dmt, dermomyotome; dmtv, ventral process of the dermomyotome; dm, dorsal muscle; dpmm, dorsal premuscle mass; int, intestine; mmp, migratory muscle precursor; pcc, precartilage condensation; pl.ompt.post, *plexus omopterygius posterior*; prn, pronephros; smp, somatopleure; vm, ventral muscle; vpmm, ventral premuscle mass

**Fig. 5:**
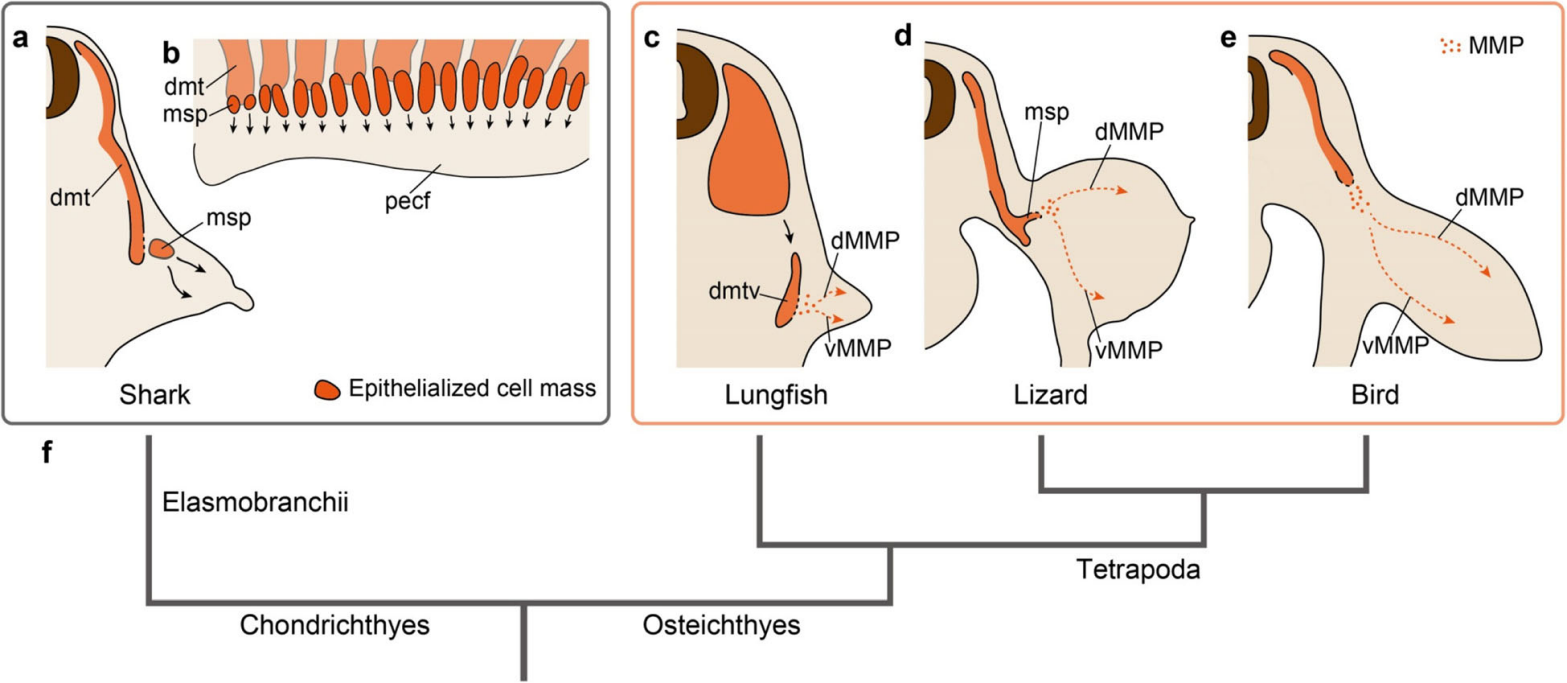
Developmental processes of the premuscle masses in the pectoral fin/forelimb buds. **a** Development of the pectoral fin premuscle masses in the shark shown in a transverse section (based on Okamoto et al. [129]). **b** Bifurcation of each pectoral fin premuscle mass in the shark in lateral view (based on Okamoto et al. [129]). **c** Development of the pectoral fin premuscle masses in the lungfish shown in a transverse section (based on Semon [99]). **d** Development of the forelimb premuscle masses in the lizard shown in a transverse section (based on Corning [112]). **e** Development of the forelimb premuscle masses in the chicken shown in a transverse section. **f** Phylogenetic relationship among taxa illustrated in (**a**–**e**). dMMP, dorsal route of migratory muscle precursors (MMPs); dmt, dermomyotome; dmtv, ventral process of the dermomyotome; msp, *Muskelsprosse*; pecf, pectoral fin bud; vMMP, ventral route of MMPs

From the evolutionary perspective, this “diffuse migration of cells into the limb” [121] seen in amniote embryos has been compared with the developmental processes of fin muscles of fishes [99, 100, 114, 122–126] (Fig. 4). In anamniotes, the dermomyotome is not often segregated from the myotome, but the corresponding structure, whose ventral part extends ventrally to develop into fin and body wall muscles, has been recognized. In shark embryos, the segmentation pattern of the somites is maintained during the development of pectoral fin muscles, as the epithelium of each myotomal sprout towards the fin bud (“*Muskelsprosse*,” “*Muskelknopsen*,” or “*muscle bud*,” in classical reports) is not dissolved until immediately before myogenesis [122, 123, 126–128]. A recent study discovered that in shark embryos the epithelium of *Muskelsprosse* is once decomposed a short time before the epithelium of segregated *Muskelsprosse* becomes recomposed [129] (Fig. 5a, b). Therefore, the fin muscles of sharks develop not from direct extension, but from the recomposed epithelialized cell mass, which is pulled apart from the dermomyotomes. In contrast, in osteichthyan fishes (sturgeon [100]; teleosts [123, 130]; and Australian lungfish [99, 131]), the epithelial structure of each myotomal extension is dissolved in the fin bud, and the *Muskelsprosse*-derived cells become mesenchymal before myogenesis (Fig. 4). With respect to this de-epithelialization of the myotomal extension, Sewertzoff [114] noted the similarity between fin muscle development in osteichthyan fishes and limb muscle development in amniotes, and suggested that the difference between osteichthyan fin and amniote limb muscles reflects solely a heterochrony of myotomal de-epithelialization (Fig. 5c–e). As others have recognized [112, 124], the position of the myotomal de-epithelialization differs proximo-distally, even among amniotes; it occurs inside the limb bud in squamates (Figs. 3 and 5d), and at the boundary between the body wall and limb bud in birds and mammals (Fig. 5e). From these lines of evidence, the developmental mode commonly observed in actinopterygian (sturgeon and teleosts) and sarcopterygian (lungfish: Figs. 4 and 5c) fishes may represent the ancestral condition for amniotes, although the evolutionary origin of the mesenchymal migration of fin/limb muscle precursor cells remains unclear. It is impracticable to infer the evolutionary relationship between the osteichthyan and chondrichthyan developmental modes (Fig. 5f), due to the lack of proper outgroup taxa, and the possibility remains that the developmental mode observed in sharks (Fig. 5a) represents a derived condition arising from the secondary loss of mesenchymal migration [129]. In the shark pectoral fin, two *Muskelsprosse* segments arise from a dermomyotome (Fig. 5b), whereas in osteichthyan pectoral fin/limb, a single *Muskelsprosse* segment arises [114]. It may be that the temporary decomposition of the epithelium of fin muscle primordium described in the shark by Okamoto et al. [129] reflects a process of *Muskelsprosse* bifurcation, which is chondrichthyan-specific.

In molecular biological studies of amniotes, both the cells undergoing diffuse migration into the limb bud and the precursor cells of hypobranchial muscle have been called “migratory muscle precursors (MMPs)” [132–136]; hypobranchial muscle precursor cells are also recognized as diffuse migrating cells in classical histological studies [112, 121, 137]. Before the initiation of myogenesis, these amniote MMPs migrate and proliferate while abutting other mesenchymal cells, which later develop into connective tissues, including bones, ligaments, and tendons. According to mouse genetics studies (reviewed by [138–141]), two transcriptional factors, namely Pax3 [142] and Lbx1 [143–145], as well as the Hgf and c-Met signaling pathway [134, 146, 147] are involved in the undifferentiated status of the MMPs. *Lbx1* gene expression has also been observed in fin/limb muscle precursor cells of anamniote gnathostomes [52, 129, 148–150]. Unlike in limb muscle and diaphragm precursor cells [133], the diffuse migration of hypobranchial muscle precursor cells (probably except for the muscles of the secondary tongue [146], which newly evolved in tetrapods [151]) does not involve the Hgf and c-Met signaling pathway [152, 153].

From the evolutionary perspective, however, this genetic regulation had not necessarily been established at the evolutionary origin of the developmental mode involving the diffuse migration of the fin/limb muscle precursor cells. Moreover, such genetic regulation is subject to developmental system drift [154]; for instance, in the axolotl, the function of *Pax3* in MMP migration is substituted by *Pax7*, allowing a gene loss of *Pax3* from the genome [155]. For these reasons, it is inappropriate to define the MMP simply by the expression of *Pax3*, *Lbx1*, and *c-Met*, when comparing fin/limb muscle development among clades broader than amniotes. Indeed, in *Lbx1*^−/−^ mice, MMPs migrate to develop into a subset of muscles [144], indicating that *Lbx1* expression is not essential for the cellular status of the MMP. Accordingly, MMP is defined as a cell that meets two criteria: (1) a mesenchymally migrating and proliferating muscle progenitor cell; and (2) a muscle progenitor cell in which differentiation is arrested.

In amniotes, the migration of MMPs begins with an intrusion into the limb bud mesenchyme, which is solely of lateral plate mesoderm (LPM) origin. In limb muscle development, MMPs are produced from the somites adjacent to the limb bud through extrinsic cues from the limb bud, although depending on the Hox code, somites can exhibit intrinsic competence to produce putative MMPs (*Lbx1*-positive cells) [156]. Observations using scanning electron microscopy and histological sections at the limb level of chicken embryos, indicate that MMP cells pass through a cell-free space above the Wolffian duct, where extracellular matrix (ECM) fibrils are accumulated [157]. Although ECM plays an important role in cell migration in general [158], its influence on the MMP colonization of the limb-level LPM remains largely unknown. In transplantation experiments, the normal migration of MMPs occurs only when they encounter the LPM at the same or earlier developmental stage [56, 159]; from this it has been inferred that the intercellular space formed by ECM becomes restricted, eventually disturbing the MMP intrusion, at later developmental stages [159].

In addition to MMPs, endothelial precursors in the limb bud are derived from somites; their cell lineage is already separate from MMPs before their intrusion into the limb bud [160–162]. Prior to MMP colonization of the limb bud, somite-derived endothelial progenitors migrate into the limb bud, which is necessary for correct MMP migration [161, 163]. The pathfinding migration of endothelial precursors into the limb bud may affect the embryonic environment such that it accepts the migration of MMPs [163], although MMPs do not precisely follow the migratory route of endothelial precursors during migration within the limb bud [164].

As described above, MMPs take two separate migratory routes, namely via dorsal and ventral masses within the limb bud [114, 165, 166] (Figs. 3 and 4). Based on transplantation experiments disturbing the order of somites, MMPs do not possess intrinsic information about their destinations [167, 168]. Indeed, each limb muscle consists of cells derived from multiple somites [164, 167, 169, 170], as predicted in classical studies [88]. Sewertzoff [114] observed that the segmental character becomes lost through a concentration of MMPs (as well as spinal nerves) at the entrance of the limb bud. The craniocaudal convergence of the limb/fin bud during its early development [171, 172] probably leads to this concentration of MMPs coming from the somites beyond the width of the limb bud.

While our knowledge about the differentiation of skeletal muscle has steadily increased [141, 173], the morphogenesis, or topographical patterning, of the limb musculature has remained relatively unclear. Nevertheless, there is compelling evidence that MMPs develop into separate muscles in response to information from the LPM [160, 174–177]. Specifically, the distribution of LPM-derived interstitial muscle connective tissue (MCT) precursors, which express *Osr1* and/or *Tcf4* transcriptional factors, mediates the myogenic regionalization, or “pre-patterning” of muscles, by providing a muscle-specific ECM and a favorable signaling environment [178–182]. In addition, it has been reported that an ectodermal signal (Wnt6) affects the myogenic regionalization [183].

During the formation of the muscle pre-pattern, molecular interactions occur between the migrating MMPs and limb mesenchyme. According to studies of chicken embryos, spatiotemporally restricted distribution of the ligand ephrin-A5 within the limb mesenchyme provides a repulsive signal for migrating MMPs, which carry the tyrosine kinase receptor EPHA4 on their cell membranes [139, 184]. The migrating MMPs also carry the CXC chemokine receptor, CXCR4, and are attracted toward the limb mesenchyme, where the CXCR4 ligand (CXCL12; also known as SDF-1) is produced [185]. CXCL12/CXCR4 signaling is involved in the secondary intrusion of limb bud-dwelling MMPs into the body wall (i.e., the in-out mechanism) [186–188].

Subsequent to the pre-patterned muscle primordia, the morphogenesis of limb muscles involves subdivision into individualized muscles (muscle splitting); each muscle is then enveloped by a continuous dense irregular MCT called the epimysium. A dense regular MCT, the tendon, attaches the epimysium to the skeletal element enabling it to transmit the muscle’s force to the skeletal element. Muscle fibers do not necessarily run parallel to tendons; in pennate muscles, for example, muscle fibers run at an angle to tendons and aponeuroses (tendinous sheets). In addition, another type of MCT, the fascia, which includes dense irregular and soft (adipose and areolar) MCTs [189], surrounds and intervenes between the epimysia and tendons.

There is compelling evidence that muscle splitting is affected by the blood vessels within the limb bud [190, 191]. In the developing limbs of chickens, the vasculature pattern is formed independent of the distribution of MMPs; muscle splitting subsequently occurs along the zone occupied by endothelial cells [191]. During this process, probably through the increased production of ECM induced by PDGFB (platelet-derived growth factor B) from endothelial cells, the MCT cells assemble at the future splitting zone, eventually subdividing the premuscle masses [191]. Whether this developmental process occurs in fin buds remains unclear, as observations have been limited to the marginal veins [100, 192]. In amniotes, blood vessels in the limb bud are composed of endothelial cells, which are differentiated from migrating somitic cells [162, 193]. Because the involvement of migrating somitic cells in the formation of the blood vessels within fin buds has not been studied in any fishes, it is impossible to determine the evolutionary origin of the migrating endothelial precursors. It should be noted that a recent detailed study of the ventral end of the dermomyotome at the pectoral fin level in shark embryos [129] did not identify any migrating endothelial precursors.

In humans, the topography of major arteries supplying the forelimb muscles shows intraspecific variation [194–198], implying that the pattern of blood vessels is not a single determinant of muscle splitting in the limb bud. Indeed, the topography of embryonic blood vessels is flexible in response to the local environment, because it is formed under the influence of oxygen and nutrient demand, as well as blood flow [199]. In the developing forelimb bud of mammals [200] and birds [201–203], a web of fine vessels (i.e., the capillary network) appears uniformly at first, and then becomes remodeled to establish branching thick vessels through poorly understood mechanisms, which may allow a certain level of variability.

Nevertheless, there is a modest evolutionary relationship between arterial and muscular topographies. In the limb-to-flipper evolution of the cetaceans, corresponding to fixations of elbow and wrist joints, the forearm and manual muscles became reduced, such that some muscles, including the biceps brachii, brachialis and intrinsic manual muscles, were lost [204–208]. Among tetrapods, cetaceans possess the simplest topography of forelimb arteries; unlike in other tetrapods, the branching of the brachial artery near the elbow joint is absent [209, 210], suggesting that loss of muscle splitting correlates with simplification of arterial topography during evolution. The evolutionary reduction of forearm and manual muscles is also recognizable in the flippers of penguins; however, major muscles are still retained as diminutive forms [211], implying that muscle splitting during embryonic development has been evolutionarily conserved. Unlike in cetaceans, the arterial topography of the penguin flipper is consistent within the range of variability of most tetrapods [211], indicating that simplification of the arterial topography may not be correlated with the decreased oxygen consumption of the muscles supplied by these arteries.

The evolutionary relationship between the arterial and muscular topographies may also be present in part in non-tetrapod sarcopterygians. In the extant coelacanth *Latimeria*, the main trunk of the pectoral fin artery bifurcates at a point just medial to the second pronator muscle [212]. Since this point corresponds to the elbow joint of tetrapods [27], the bifurcating arteries are likely homologous with the radial and ulnar arteries of tetrapods.

Muscle splitting in the forelimb bud is not identical to that in the hind limb bud, although both limb muscles develop from similar premuscle masses. In studies of chicken [213, 214] and mouse [215, 216], a paired-type homeodomain transcriptional factor Pitx1 is responsible for the morphological identity of the hind limb, and misexpression of *Pitx1* in the LPM-derived forelimb bud mesenchyme results in homeotic transformation from forelimb- to hind limb-like muscle patterns. A similar homeotic transformation has also been identified in a human congenital anomaly, Liebenberg syndrome, the etiology of which involves a genomic change at the *PITX1* locus [217]. These studies on *Pitx1* suggest that limb muscle patterns emerge in accordance with information within the limb bud mesenchyme prior to the migration of the MMPs.

Although muscle splitting plays a central role in the patterning of limb muscles, it should be noted that individual muscles are not always formed directly through the muscle splitting of a premuscle mass [218]. For example, after muscle splitting, multiple muscle primordia fuse into a single muscle (secondary fusion) during the development of pectoralis and brachialis muscles in humans [218]. Furthermore, during development of the human extensor digiti minimi muscle, primordia are formed at the fourth and fifth digits; however, the primordium at the fourth digit later disappears [218]. These secondary remodeling processes of developing muscles are indispensable to the formation of taxon-specific muscle topography.

During the development of individual muscles, multiple myoblasts fuse to form multinucleated myotube cells [219]; subsequently additional myoblasts fuse to the myotube, eventually forming myofiber cells [220]. Satellite cells, which are derived from the shared cell population with myoblasts, are also incorporated in each muscle and reside between the sarcolemma and basement membrane of myofibers [141, 221].

The process of limb muscle regeneration shows similarities with the developmental process. In amniotes, satellite cells proliferate and differentiate into myoblasts during skeletal muscle regeneration [141, 221–223]. At the differentiation from the satellite cell to myoblast, *Lbx1* is transiently expressed [224], reminiscent of the differentiation from the MMP to myoblast in embryonic development. Subsequently, differentiating satellite cells migrate to the regenerating site while interacting with MCT expressing *Tcf4* [223]. Ephrins produced by neighboring myofibers also likely provide repulsive signals for migrating satellite cells [222]. These satellite cell behaviors are suggestive of commonality with MMP migration in the limb buds.

As mentioned earlier, urodeles are able to regenerate an amputated limb, and the topography of limb muscles is recurrently formed during this process [67, 225]. In urodeles, satellite cells are the source for regenerated limb muscles before metamorphosis; however, after metamorphosis, regenerated limb muscles are derived from de-differentiated myofiber cells [85, 86, 226]. Although the mechanism of the recurrent generation of individual muscles in an amputated limb remains unclear, it may be important for understanding how limb muscle homology is maintained during evolution.

## Interaction between developing muscles and tendons in amniotes

The tendon is a dense, highly organized fibrous connective tissue, composed predominantly of type I collagen, which transmits a uniaxial force between a bone and a muscle [181, 227, 228]. It is very similar to the ligament, which is connected solely to bones [229]. At the junction between the bone and the tendon or ligament, there is a transitional tissue, or fibrocartilage, in which chondrocytes are enclosed, and the fibers of tendon or ligament connect to the periosteum [230]. In the development of this junction, a common progenitor cell population that co-express *Sox9* and *scleraxis* (*Scx; a basic helix-loop-helix (bHLH) transcriptional factor*) differentiates into tendon or ligament cells (tenocytes or ligamentocytes, respectively) or chondrocytes at the attachment site of the bone (enthesis) [231–233]. However, the patterning of the tendon is not necessarily coupled with that of the skeleton; rather it forms under shared cues with the muscle patterning [234]. On the muscle side, the fibers of the tendon connect to the epimysium and perimysium; i.e., the MCTs surrounding the individual muscles and bundles of myofibers, respectively. The junction between the muscle and tendon initially form as a specialized region of the epimysium and later become contiguous with the perimysium [235].

The formation of individual muscles in the limb is intimately related with tendon development. According to the experiments focusing on tendon development in the absence of muscle, and vice versa, in chicken embryos, the differentiation of tendon and muscle progenitors occur independently of each other, but the subsequent muscle splitting and segregation of tendon primordia into individual tendons require reciprocal interactions between the developing muscle and tendon [236]. These interactions involve Fgf signaling in chicken embryos [237–239]. Such interactions are responsible not only for the topography of the muscles, but also for the shape of muscle bellies sculptured by myofiber apoptosis [240]. In mice, tendons in the limb, in particular in the zeugopod (forearm), become elongated as the zeugopod skeleton elongates, after the establishment of the connection between muscle and tendon [241]. Late in this morphogenetic process, the flexor digitorum superficialis muscles, which initially develop in the autopod (manus), become translocated into the zeugopod [241, 242].

Our current understanding of tendon development is based largely on studies of Scx, which is a specific marker for tendons and ligaments [238, 243–248]. Although some signaling molecules, including transforming growth factor β (Tgfβ) and CXC chemokines, likely regulate differentiation and maintenance of *Scx*-expressing tendon progenitors [249, 250], how the tendon progenitors are specified in embryonic mesenchyme remains unsolved.

## Interaction between developing muscles and motoneuron axons in amniotes

Although the evolutionary conservation of the topography of the brachial plexus and peripheral branching axons has attracted the attention of researchers in comparative anatomy, the morphogenesis of limb-innervating nerves remains for the most part unclear.

**Fig. 6:**
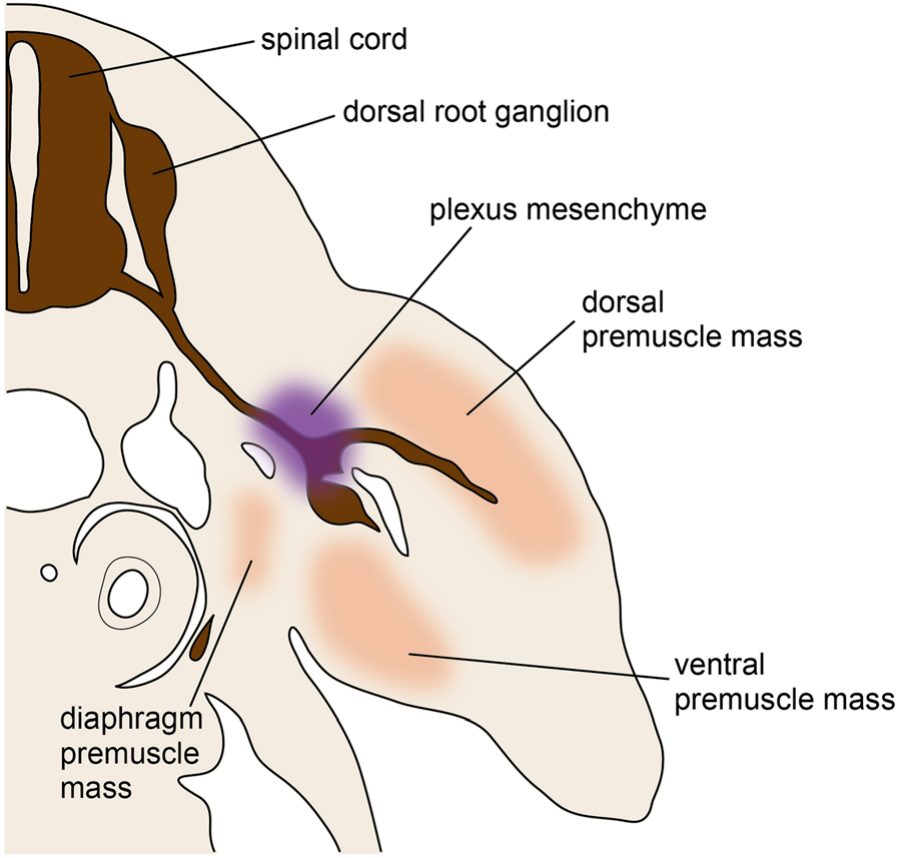
Schematic drawing of the plexus mesenchyme in the E10.5 mouse. Based on Wright and Snider [251]. The plexus mesenchyme expressing *Gdnf* is of lateral plate mesodermal origin, and does not involve muscle precursor cells

In embryonic development, the brachial plexus is formed at the “plexus mesenchyme” [251] (Fig. 6), which consists of LPM at the base of the limb bud. *Gdnf* (*glial cell line-derived neurotrophic factor*) is transiently expressed in the plexus mesenchyme, and likely supports neurons while their axons organize in the plexus [251–253]. The migrating MMPs are diverged into the dorsal and ventral premuscle masses at the plexus mesenchyme [251], suggesting that the plexus mesenchyme also affects the MMP migration. In addition, the fact that the development of the latissimus dorsi and cutaneous maximus muscles, both of which develop through the in-out migration of MMPs from the limb bud, requires *Gdnf* after the formation of the plexus [252] should be noted in light of the involvement of the plexus mesenchyme in MMP migration.

The timing of the first contact between nerve axons and developing muscles varies among tetrapods. In mammals and anurans, nerve axons enter the limb premuscle masses almost concurrent with premuscle mass formation [254–256], while in birds the axons remain at the plexus region prior to the onset of primary myotube formation [257, 258]. Considering this interspecific difference, interaction between the nerve axon and developing muscle may not be required for the morphogenesis of limb muscles before the primary myotube formation.

**Fig. 7:**
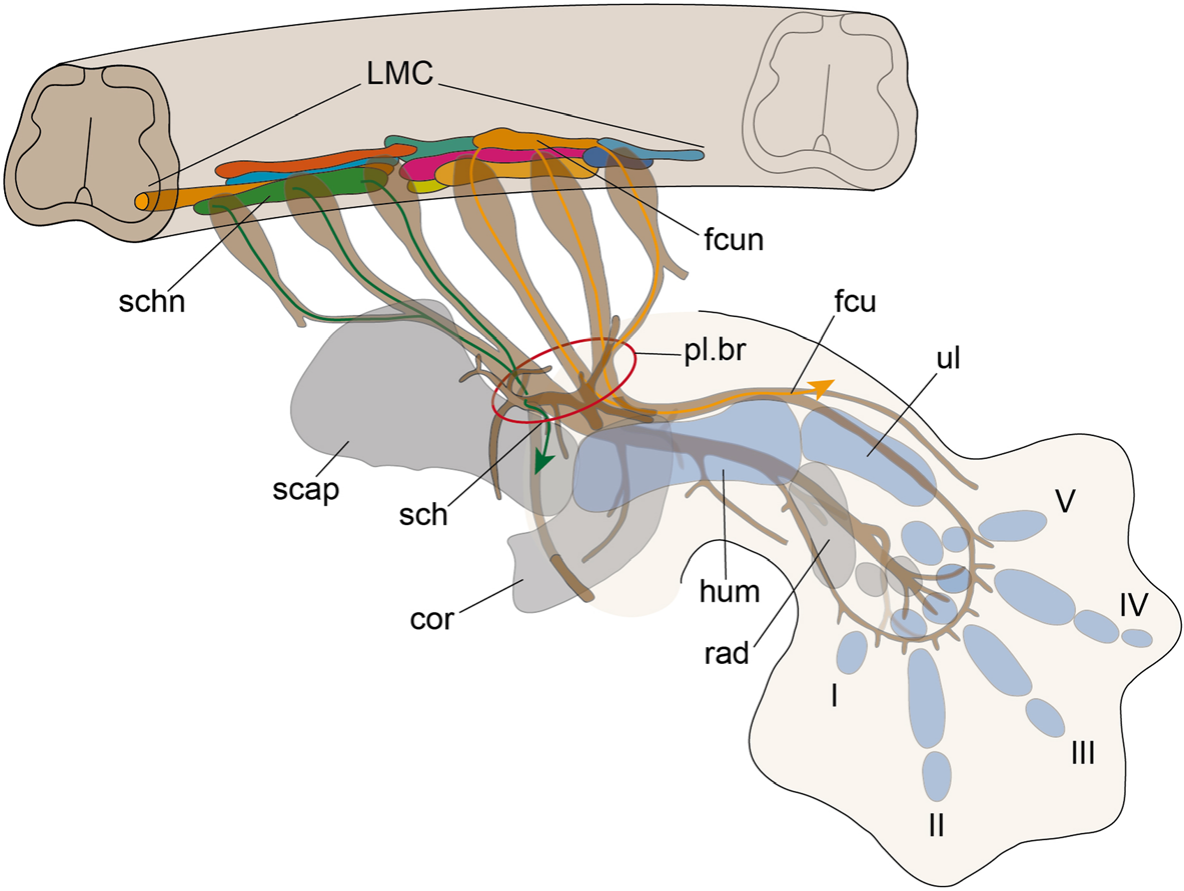
Columnar organization of motor neuron pools and the topography of nerves innervating limb muscles in tetrapods. Motoneuron columnar organization is based on the study using the chicken [259]. Topography of nerves and skeletal elements of *Tarentola* (*Ascalabotes*) *fascicularis* (gecko) illustrated in Sewertzoff [114] is shown as a representative. Skeletal elements of the metapterygial axis are colored in blue, and the other (preaxial) skeletal elements in gray. Nerves for the scapulohumeralis (green) and flexor carpi ulnaris (orange) muscles are shown as examples of correspondences between motoneuron pools and their respective target muscles. cor, coracoid; fcu, nerve for the flexor carpi ulnaris muscle; fcun, motoneuron for the flexor carpi ulnalis muscle; hum, humerus; scap, scapula; I–V, digits I–V; LMC, lateral motor column; sch, nerve for the scapulohumeralis muscle; schn, motoneuron for the scapulohumeralis muscle; pl.br, *plexus brachialis*; rad, radius; ul, ulna

The limb-innervating motoneurons are specified according to the expression pattern of Hox genes in the spinal cord [259–264]. Although the limb muscle-innervating motoneurons are specified at the level of MMP-producing somites along the body axis, experimental perturbations have indicated that the specification of these motoneurons are independent of those of MMP-producing somites [265]. The axons of the limb muscle-innervating motoneurons extend to innervate the corresponding muscles (Fig. 7) in accordance with the surrounding environment, as shown by experimental perturbations of avian embryos [177, 266–269]. The correspondence between the motoneurons and each muscle (Fig. 7) is determined prior to the innervation, as exemplified by experiments displacing motoneuron pools in the chicken embryo by craniocaudally reversing the spinal cord at the lumbosacral level [266], as well as by perturbing the Hox code in the spinal cord at the brachial level [259].

Detailed observation of the chicken hind limb has shown that, in the limb mesenchyme, the axons pass across the domain where glycosaminoglycans are thin, so the axons do not pass the domains where cartilages later develop [270]. Similarly, a recent study of various amniote embryos suggested that a class 3 semaphorin, Sema3A, secreted from chondrocytes provides a repulsive signal for axonal guidance [271]. In contrast, β-catenin stabilization in muscle provides an attractive signal to the axons [272, 273]. Considering the hypothesis of Fürbringer [88] regarding the evolutionary change that brought about the brachial plexus, the possibility that the pathfinding of the axons follows the regionalization associated with the metapterygial axis in the limb mesenchyme (Fig. 7) deserves consideration.

In vertebrates, at the junction between a motoneuron axon and muscle, there is a specific type of synapse, namely the neuromuscular junction, in which acetylcholine (ACh) functions as an excitatory neurotransmitter to cause muscle contraction [274]. Prior to the arrival of the motoneuron axon, ACh receptors (AChRs) are aggregated to form multiple clusters (aneural AChR clusters) at a region in the middle of the myofibers. This AChR-aggregated area is the foundation of the neuromuscular junction, in that the nerve terminals arrive at certain aneural AChR clusters to initiate synaptogenesis [274]. Until the completion of the neuromuscular junction formation, which proceeds postnatally for 2 weeks in the mouse, a single myofiber is transiently innervated by axons of multiple motoneurons, although it later becomes innervated by only a single motoneuron axon through reciprocal interactions between the muscle and synapse plus terminal Schwann cells [275–279].

Experiments involving removal of nerves in the chicken embryo have demonstrated that interaction with nerves is not responsible for muscle splitting [280, 281]. In contrast, neuromuscular junction formation is involved in the later phase of development after the formation of primary myotubes, as contractions of muscles are responsible for the morphogenesis of muscles [250, 282, 283] and the bony ridges at muscle attachment sites [284].

## Observed variability in the forelimb muscles

The developmental process explained above allows modest intraspecific variability in morphology of forelimb muscles. Since forelimb muscles are present as paired structures, intraspecific variations showing fluctuating asymmetry [285, 286] are expected to originate from developmental fluctuation rather than the genetic background. Indeed, such variations have been reported in human anatomy; e.g., the muscular axillary arch [*Muskulöser Achselbogen*] and sternalis muscle as variations of the pectoralis muscle [287–295]. In another case, extensive fluctuating asymmetry has been reported for wing muscles of the flightless bird, emu (*Dromaius novaehollandiae*), suggesting relaxed stability of the developmental mechanism in the vestigial limb [296]. Future studies of the variability observed in limb muscles may improve our understanding of the relationship between developmental fluctuation and evolvability.

## Diaphragm: an evolutionary novelty of the mammalian lineage

**Fig. 8:**
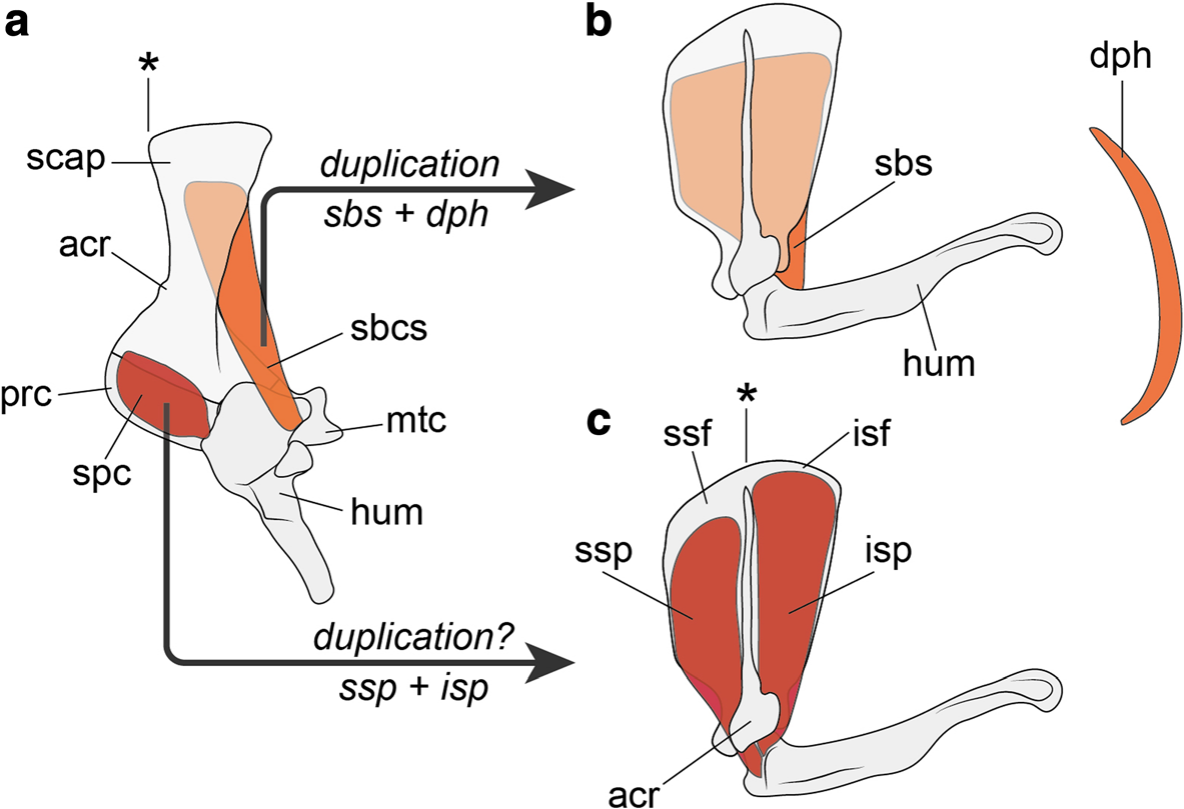
Putative evolutionary origin of the diaphragm through a partial duplication of MMP population. Based on Hirasawa and Kuratani [110]. Left lateral views. **a** Subcoracoscapular muscle (subscapular muscle-homolog) of the dorsomedial forelimb muscle group and the supracoracoid muscle of the ventrolateral forelimb muscle group in a Pelycosaur-grade taxon, *Dimetrodon* (based on Romer [19]). **b** Subscapular muscle and the diaphragm, which evolved from the subcoracoscapular muscle of Pelycosaur-grade ancestors, in an extant mammal, *Didelphys* (based on Jenkins and Wejs [304]). **c** Supraspinatus and infraspinatus muscles in *Didelphys*. Note the cranial margin of the scapula (*) in (**a**) corresponds to the border between the supraspinatus and infraspinatus fossae (*) in (**c**) [117, 305]. acr, acrominon; dph, diaphragm; isf, infraspinatus fossa; isp, infraspinatus muscle; hum, humerus; mtc, metacoracoid; prc, procoracoid; sbcs, subcoracoscapular muscle; sbs, subscapular muscle; scap, scapula; spc, supracoracoid muscle; ssf, supraspinatus fossa; ssp, supraspinatus muscle

As mentioned above, after their evolutionary origin, forelimb muscles have evolved without drastic modification. However, recent studies of evolutionary changes in forelimb position along the body axis have found that the mammalian diaphragm likely evolved from a shoulder muscle, through a partial duplication of the forelimb MMP population [110, 297]. In particular, comparison of brachial plexuses among amniotes suggests that the diaphragm evolved from the subscapular muscle of the ancestor [110] (Fig. 8a, b). In the evolution toward mammals, the supracoracoid muscle diverged into two muscles, namely the supra- and infraspinatus muscles [19], and it is possible that the evolutionary origin of these two muscles coincided with that of the diaphragm through a divergence from the ancestral subscapular muscle (Fig. 8a, c).

Some classical papers of comparative anatomy [298, 299] have suggested that the diaphragm evolved from the rectus cervicus muscles, namely hypobranchial muscles, which also develop from MMPs. However, recent studies of developmental biology have highlighted commonalities between the diaphragm and forelimb muscles: both these muscles develop in LPM-derived mesenchyme expressing *Tbx5* and *Hgf*, unlike the hypobranchial muscles [119, 133, 152, 153]. In addition, a few clinical cases of associated movements between the diaphragm and some forelimb muscles (Erb’s palsy) in patients who experienced birth injuries of the brachial plexus have been reported [300–302]. As suggested by Oosuga [303], it is possible that these cases reflect the forelimb muscle-like identity of the diaphragm.

As a candidate exception to forelimb muscle homology, the diaphragm offers an exclusive opportunity for understanding when and how a drastic modification was possible in the evolutionary history after the establishment of an evolutionary novelty, or a new developmental constraint.

## Conclusions

1. At the pectoral fin-to-forelimb transition, the number of muscles increased, while the number of spinal nerves innervating these muscles decreased. The brachial plexus is an evolutionary novelty of tetrapods. Within the tetrapod lineage, limb muscle homology has been largely conserved.
2. Forelimb muscles develop from diffuse migrating somitic cells, or MMPs. The limb muscle homology is generated mainly through subdivision of myoblast masses (muscle splitting). The LPM-derived limb mesenchyme likely provides the information for the proper distribution of MMPs, and subsequently the MCTs differentiated from the LPM-derived limb mesenchyme subdivide each myoblast mass into individual muscles. Development of blood vessels plays some role in the latter process.
3. The reciprocal interaction with tendon progenitors is necessary for the morphogenesis of individual muscles, but the tendon and muscle progenitors begin their differentiation independently of each other.
4. Although the topography of the brachial plexus and the relationship between the nerves and their innervating forelimb muscles are evolutionarily conserved, the developmental mechanism recurrently generating them remains largely unclear, and should be the subject of future analyses.
5. In addition to further studies on the developmental mechanism recurrently generating the forelimb muscle homology, particularly focusing on MCTs and tendons, studies on intraspecific variability of the forelimb muscle morphology and research on the diaphragm as a putative derived forelimb muscle will lead to our better understanding of the role of developmental constraints in evolution.
